# Decreased Yes-Associated Protein-1 (YAP1) Expression in Pediatric Hearts with Ventricular Septal Defects

**DOI:** 10.1371/journal.pone.0139712

**Published:** 2015-10-01

**Authors:** Lincai Ye, Meng Yin, Yu Xia, Chuan Jiang, Haifa Hong, Jinfen Liu

**Affiliations:** 1 Department of Thoracic and Cardiovascular Surgery, Shanghai Children’s Medical Center, Shanghai Jiaotong University School of Medicine, Shanghai, China; 2 Institute of Pediatric Translational Medicine, Shanghai Children’s Medical Center, Shanghai Jiaotong University School of Medicine, Shanghai, China; 3 Shanghai Pediatric Congenital Heart Disease Institute, Shanghai Children’s Medical Center, Shanghai Jiaotong University School of Medicine, Shanghai, China; 4 Department of Thoracic and Cardiovascular Surgery, Xinhua Hospital, Shanghai Jiaotong University School of Medicine, Shanghai, China; Cedars Sinai Medical Center, UNITED STATES

## Abstract

**Background:**

Ventricular septal defects (VSDs) are the most common and simplest type of congenital heart diseases (CHDs). Animal studies have suggested that the downregulation of Yes-associated protein 1 (YAP1) during embryonic development causes VSD-associated CHDs. However, how YAP1 contributes to isolated VSD (iVSD) is unclear.

**Methods and Results:**

Twenty right atrial specimens were obtained from iVSD patients during routine congenital cardiac surgery and we assessed YAP1 expression in these specimens. For controls, six right atrial specimens were obtained from normal hearts of children without heart disease, three of whom died from cerebral palsy, and three who underwent heart transplants. YAP1 mRNA and protein levels and nuclear localization were significantly reduced in iVSD specimens compared to normal heart specimens. Concomitantly, mRNA levels of *YAP1* downstream targets *CTGF* and *AXL* were also significantly decreased in iVSD specimens. Although Ki67-positive cardiomyocytes in iVSD specimens were comparable to normal heart specimens, Ki67-positive non-cardiomyocytes were significantly decreased.

**Conclusions:**

YAP1 expression was markedly decreased in hearts of iVSD children. Given the important role of YAP1 during heart development, downregulation of YAP1 expression may contribute to iVSD and affect the proliferation of non-cardiomyocytes.

## Introduction

Congenital heart diseases (CHDs) are the most common type of birth malformations. Although recent advances in pre- and neonatal diagnoses and surgical procedures have reduced the morbidity and mortality for many CHDs, the etiologies of these conditions are still undefined [1–3]. Studies of ventricular septal defect (VSD), the most frequent and simplest form of CHD, might offer novel insights into the etiology of CHD. VSDs arise from one of three causes: tissue/planar cell polarity (PCP) driving directed growth and fusion of two tissue masses [4], endothelial- to- mesenchymal transition (EMT) of the atrioventricular cushion [5], and proliferation of cardiomyocytes and mesenchymal cells [6].

YAP1, a key effector of the hippo signaling pathway [7], has been suggested to be pivotal in EMT [5] and cell proliferation [6] in mammalian embryonic heart development. When a conditional YAP1 allele (YAP1^flox^) in cardiomyocytes was inactivated early in heart development using Tnnt2–Cre [8], mice had severe VSD with markedly hypoplastic myocardium [6]. In addition, when a conditional YAP1 allele (YAP1^flox^) in endothelial cells was inactivated using Tie2-Cre [9], there was a hypocellular cushion defect due to EMT failure [5]. Until now, there was no isolated VSD (iVSD) [10] animal model, therefore animal studies have been complicated by other forms of CHDs. Therefore, iVSD samples obtained from human heart tissue would be beneficial in uncovering the role of YAP1 in ventricular septum formation.

Here we assessed YAP1 expression in postnatal iVSD patients, and found a significantly lower expression in the hearts of iVSD patients compared to normal hearts. In examining Ki67 expression, a marker of cell proliferation, the number of Ki67-positive cardiomyocytes in the postnatal period in iVSD was not significantly different from those in normal hearts; while interestingly the number of proliferating non-cardiomyocytes was significantly reduced in iVSD. These results suggest that lower YAP1 expression may contribute to the formation of iVSD and affects the closure of iVSD during the postnatal period by decreasing the proliferation of non-cardiomyocyte cells.

## Materials and Methods

### Study population and tissue sampling

Right atrial tissues (0.2 cm × 0.2 cm × 0.3 cm) from the same heart region near the operation incision were collected from twenty iVSD patients at the Shanghai Children’s Medical Center between September 2014 and June 2015. Six normal right atrial tissues were collected from children who died of cerebral palsy or from heart transplant tissues. Each tissue was preserved in liquid nitrogen and later divided into four parts and used for Western blot, qRT-PCR, immunofluorescence, and flow cytometry analyses. The Animal Welfare and Human Studies Committee at the Shanghai Jiaotong University School of Medicine approved all procedures, and parental written informed consent was obtained prior to study initiation.

### Western blot

Immunoblotting was used to measure YAP1 expression. Briefly, individual atrial tissues were solubilized in Laemmli buffer containing 2-mercaptoethanol, and proteins (20μg/lane) were separated on 10% SDS polyacrylamide gels. After proteins were transferred onto polyvinylidene fluoride membranes (Merck, Millipore, Billerica, MA), membranes were blocked with 5% non-fat milk in Tris-buffered saline with Tween 20 (TBST) for 2h at room temperature. Blots were then probed with the following antibodies at 1:1,000–2,500 dilutions: anti-YAP1 (ab52771, Abcam, Hong Kong) and anti-GAPDH (ab8245, Abcam). Dylight 800- labeled affinity secondary antibodies (072-07-15-06, Kirkegaard & Perry Laboratories, Gaithersburg, MD) were then used. Quantitative densitometry image analysis normalized to GAPDH was performed using Image J (NIH).

### Immunofluorescence

Immunofluorescence was used to confirm Western blot data. Seventy-five consecutive cryosections were prepared on 15 slides. The initial slide was selected using a random number generator, and then every fifth subsequent slide was selected for staining. The slides were blocked using 10% FBS for 30 min, and then incubated with the following antibodies at 1:200 dilutions: rabbit anti YAP1 (ab52771, Abcam), and mouse anti-troponin T (ab8295, Abcam) at room temperature for 2 h. The slides were then incubated with the following secondary antibodies: Fluor® 555-conjugated anti-mouse (Abcam, ab150107; 1:1,000 dilution), and Alexa Fluor® 488-conjugated anti-rabbit (Abcam, ab150073; 1:1,000 dilution). Cell nuclei were stained with DAPI.

### Real-time quantitative PCR analysis

Total RNA was extracted with Trizol (Invitrogen) and PrimeScriptTM reagent kit (Takara, Japan) was used for RT-PCR. Quantitative real-time PCR (qRT-PCR) reactions were carried out using SYBR Green Power Premix Kits (ABI) according to the manufacturer’s instructions. The qRT-PCR reactions were performed with an Applied Biosystems 7500 Fast Real-Time PCR System and the following conditions: 1 cycle of 10 s at 95°C, followed by 40 cycles of 15 s at 95°C and 60 s at 60°C, according to the manufacturer’s instructions. The primers were obtained from Generay Bio (China). The sequences were as follows: YAP1(177 bp) sense primer, 5’- TAGCCCTGCGTAGCCAGTTA-3’and antisense primer, 5’- TCATGCTTAGTCCACTGTCTGT-3’; GAPDH(87 bp) sense primer, 5’-TGCACCACCAACTGCTTAGC-3’and antisense primer, 5’-GGCATGGACTGTGGTCATGAG-3’; CTGF(207 bp) sense primer, 5’-TACCAATGACAACGCCTCCT-3’and antisense primer, 5’-TGTGGAGTATGTACCGACGG-3’; AXL(180 bp) sense primer, 5’-TTTACAGAGCTGCGGGAAGA-3’and antisense primer, 5’-AGGATTCCTGTAGCTGCCTC-3’.

### Single cell suspensions of iVSD and normal hearts

Myocardial samples stored in liquid nitrogen were thawed in ice-cold PBS for 10min, cut into 1- mm^3^ blocks, and washed twice in solution A (120 mmol/L NaCl, 5.4 mmol/L KCl, 5 mmol/L MgSO_4_, 5mmol/L pyruvate, 20 mmol/L glucose, 20 mmol/L taurine, 10 mmol/L HEPES, 5 mmol/L nitrilotriacetic acid pH 7.4). Samples were then incubated in solution B (120 mmol/L NaCl, 5.4 mmol/L KCl, 5mmol/L MgSO_4_, 5 mmol/L pyruvate, 20 mmol/L glucose, 20 mmol/L taurine, 10 mmol/L HEPES, 0.05 mmol/L CaCl_2_, 0.4 mg/mL collagenase type II, Sigma,C6885) for 40 minutes. Every 10min dissociated cells were collected in 10% FBS/DMEM-F12 (vol/vol), and 5 ml of solution B added to digest remaining tissues.

### Flow cytometry analysis

Isolated cardiac cells were stained with mouse monoclonal antibodies against cardiac troponin T (Abcam, ab8295, 1:200 dilution) and Alexa Fluor® 488-conjugated rabbit anti-Ki67 antibodies (Abcam, ab154201; 1:200 dilution) overnight. After three washes, the cardiac cells were incubated with Alexa Fluor® 647-conjugated anti-mouse secondary antibodies (Abcam, ab150107; 1:1,000 dilution). After three washes the stained cardiac cells were analyzed using a BD FACSAria cell sorter. Three independent experiments were performed for each analysis.

### Laser scanning confocal microscopy analysis for Ki67 expression

iVSD and control heart tissue sections were used for Ki67 staining. The initial slide was selected using a random number generator, and every seventh slide after that was chosen for microscopy in a random-systematic fashion. The slides were stained with mouse monoclonal antibodies against cardiac troponin T (Abcam, ab8295, 1:200 dilution) and Alexa Fluor® 488-conjugated rabbit anti-Ki67 antibodies (Abcam, ab154201; 1:200 dilution) overnight. After three washes, the sections were incubated with Alexa Fluor® 555-conjugated anti-mouse secondary antibodies (Abcam, ab150107; 1:1000 dilution). Three researchers who were blinded to sample identity quantified the cellular Ki67 events using either manual counting or digital thresholding (image segmentation and the creation of a binary image from a gray scale). Analysis of the converted binary images was performed using Image Processing and Analysis in Java software (Image J).

### Statistical Analysis

Continuous data, including age, weight, protein expression, mRNA levels and number of Ki67-positive cell, were expressed as mean ± standard deviation. Differences were tested by two-tailed Student’s *t*-test. Categorical variables were expressed by count and percentage, which were compared between survival and death by the Fisher’s exact test. P-values < 0.05 were considered statistically significant. Statistical analyses were performed using SAS software version 9.2 (SAS Institute Inc., Cary, NC, USA).

## Results

### Baseline patient characteristics

Twenty infants with iVSD hearts (S1 Table) and six subjects with normal hearts were included in the study. No differences in age and gender were observed between the two groups (Table 1). However, the body weight was significantly lower in the iVSD group as compared to the normal heart group (*p* = 0.006, Table 1), which was likely due to feeding difficulties. The average diameter of VSD was 1.1±0.14 cm to require surgical closure. The patient characteristics were well balanced and suitable for studying the cause of iVSD.

**Table 1:** Clinical information of the iVSD patients and normal children.

	iVSD heart (n = 20)	Normal heart(n = 6)	*p*
Weight (kg)	6.9±1.6	7.4 ± 1.4	0.006(**)
Age (months)	6.0±1.7	5.6±0.6	0.44
Gender (male/female)	12/8	4/2	0.68
Diameter of VSD(cm)	1.0±0.16	-	-
Type of VSD	Perimembranous	-	-

** Differences were tested by two-tailed Student’s *t*-test.

### YAP1 protein expression is decreased in atrial tissue derived from iVSD patients

**Fig 1:**
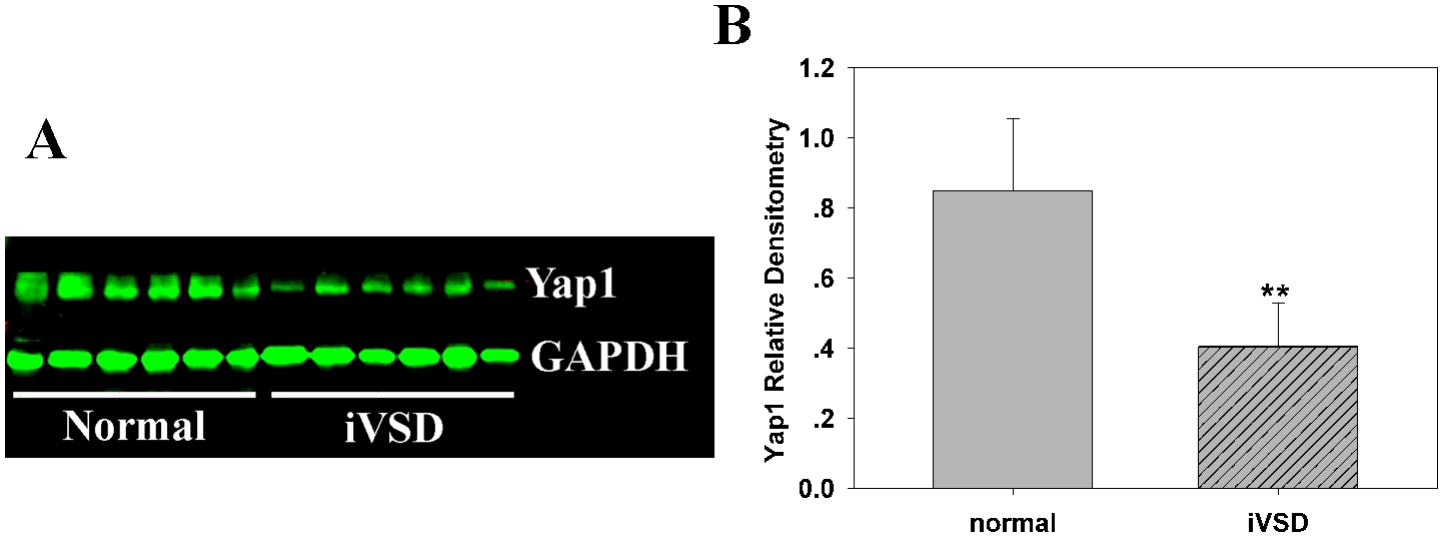
YAP1 protein expression is decreased in atrial tissue derived from iVSD patients. (A)Western blot analysis of resolved atrial tissues from control and iVSD hearts demonstrates that YAP1 is significantly decreased in iVSD hearts. (B) Numeric data obtained by densitometry analysis using GAPDH as a loading control. Data presented as means ± SDs. Student’s *t*-tests were performed to evaluate statistical significance (iVSD group: *n* = 20; control group: n = 6); **p < 0.01.

To analyze YAP1 protein levels, tissue extracts from iVSD (n = 20) and normal atria (n = 6) were analyzed using Western blots. The results demonstrated that YAP1 protein levels in iVSD hearts were significantly decreased compared with normal hearts (Fig 1A and 1B; *p* < 0.01).

### Immunofluorescence staining of YAP1 expression in iVSD patients

**Fig 2:**
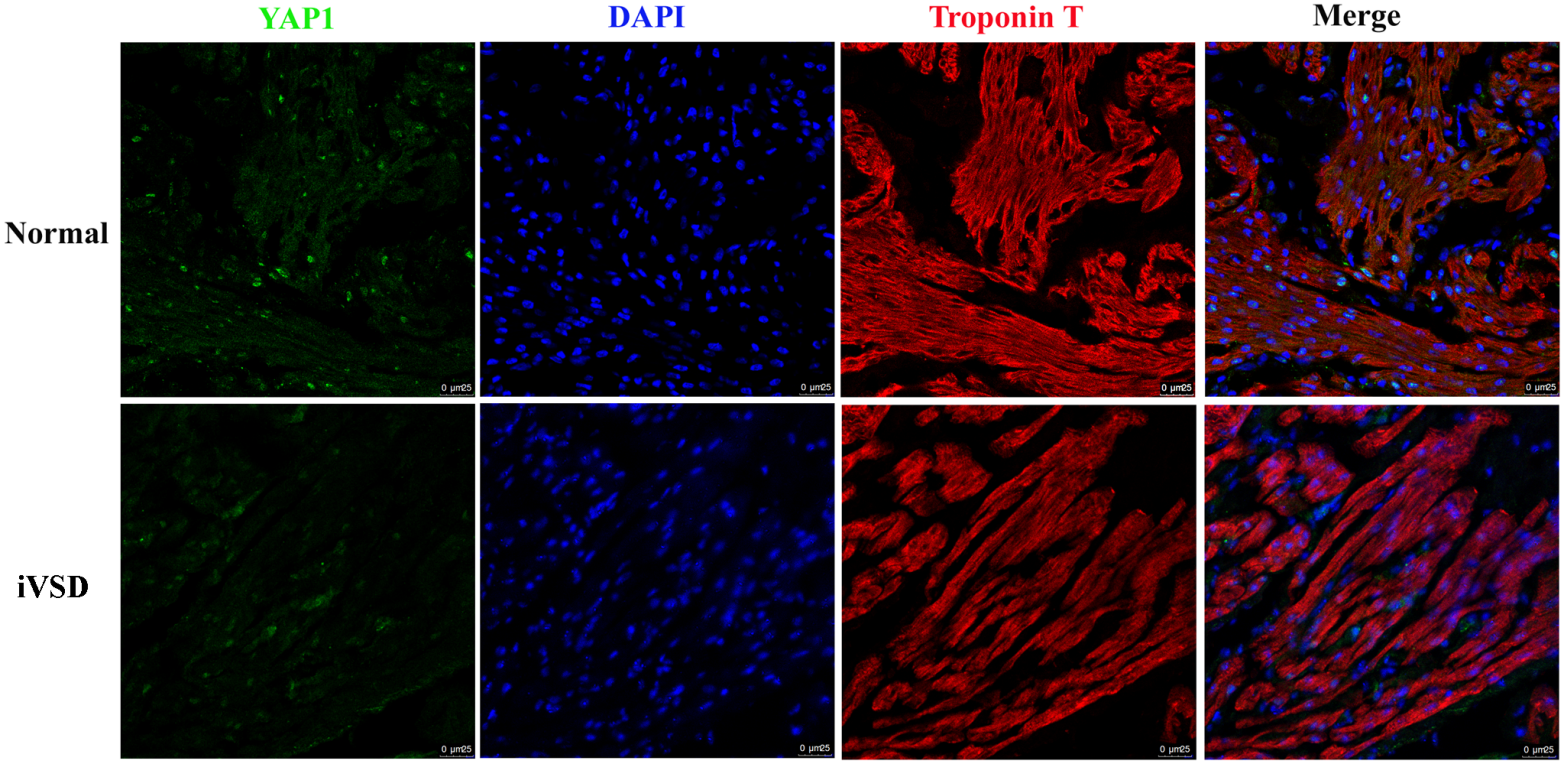
Immunofluorescence revealed that YAP1 expression was significantly lower in iVSD hearts compared to control hearts. Cardiac troponin T (red), YAP1 (green), and DAPI (blue) staining are shown; Scale bar = 25μm.

To confirm the results from the Western blot and to determine the cellular origin of YAP1, iVSD (n = 20) and normal tissue (n = 6) sections were stained using cardiac troponin T (a specific marker for cardiomyocytes) in combination with anti-YAP1 antibodies. The immunofluorescence results confirmed that Yap1 protein was significantly decreased in iVSD tissues. In addition, YAP1 was expressed both in cardiomyocytes and non-cardiomyocytes and YAP1 protein was localized chiefly in the nucleus (Fig 2).

### Messenger RNA levels of YAP1 and its downstream targets are decreased in iVSD patients

**Fig 3:**
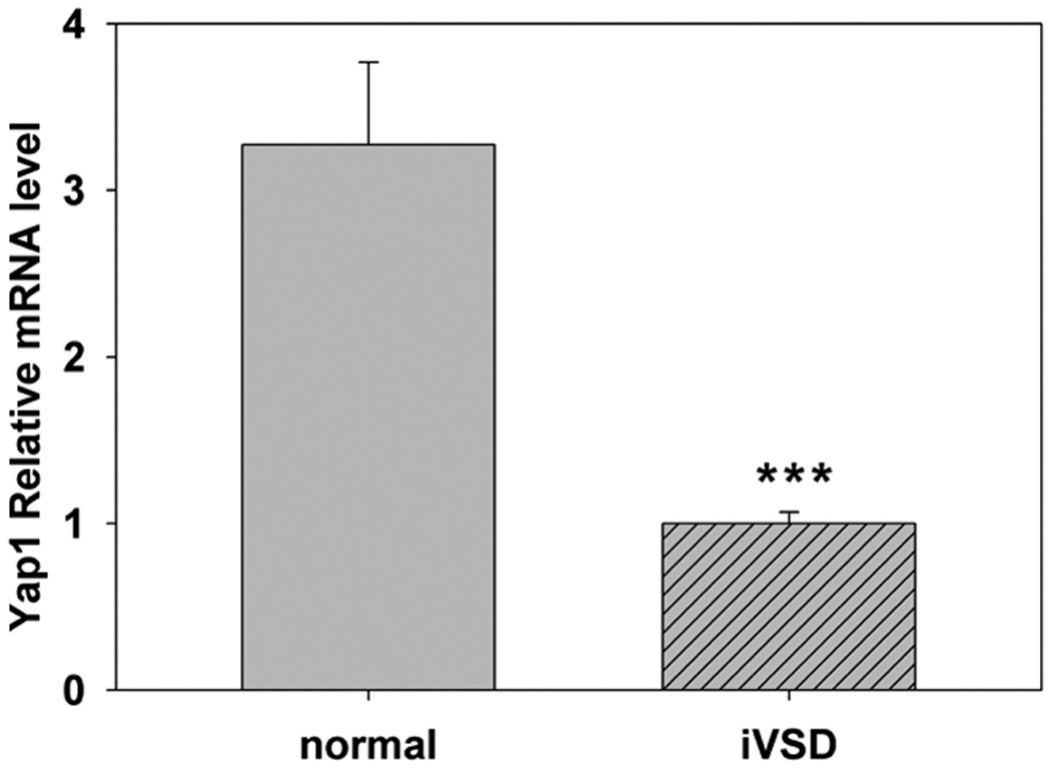
*YAP1* mRNA is significantly lower in iVSD hearts compared to control hearts. Quantitative RT-PCR was used to analyze message levels of YAP1 in iVSD and control hearts. Our results indicate that *YAP1* is significantly decreased in iVSD samples compared to normal controls. *GAPDH* served as a control. Bars indicate means ± SD. The Student’s *t* _test was performed to evaluate statistical significance, iVSD group: *n* = 20; control group: n = 6, ***p<0.001.

To assess whether there was a decrease in YAP1 mRNA expression, total RNA from both iVSD and normal hearts was extracted and measured using qRT-PCR analysis. The results showed that *YAP1* relative mRNA levels (Fig 3) in iVSD hearts were decreased compared to normal hearts (1.03±0.0674 in iVSD hearts compared to 3.274±0.4951 in normal hearts, *p*<0.001; normal heart/iVSD heart ratio = 3.074). The results from the qRT-PCR analysis indicated that *YAP1* gene expression was significantly reduced in iVSD patients resulting in lower protein levels and suggested that the decrease in YAP1 was associated with the incidence of iVSDs in patients.

**Fig 4:**
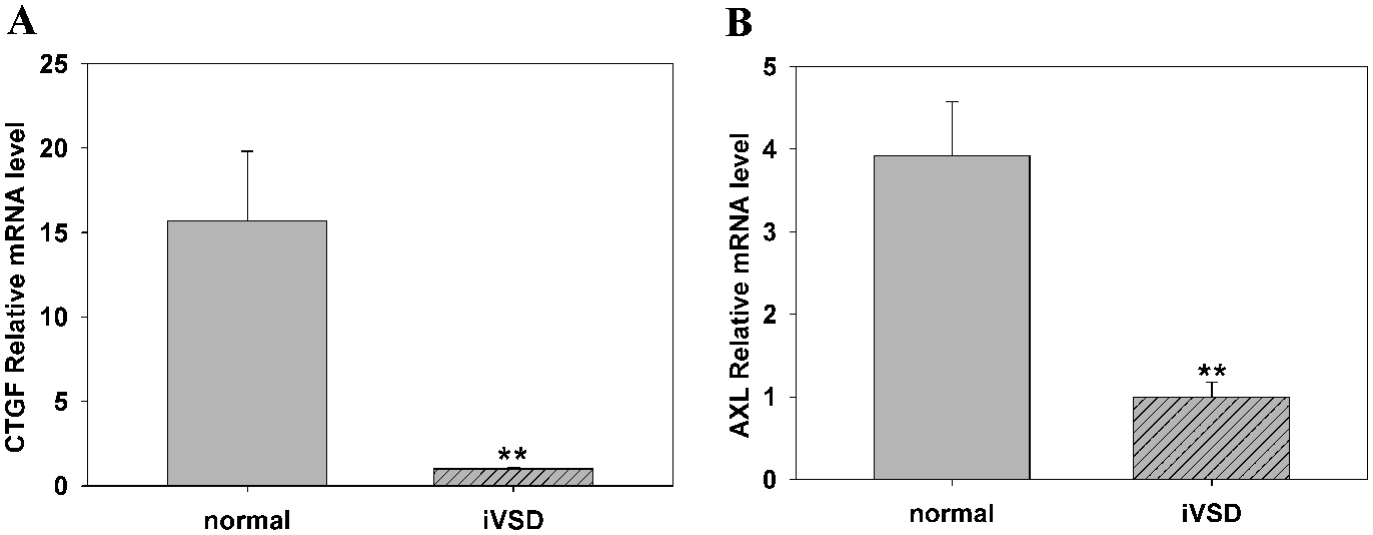
Downstream targets of *YAP1* are significantly lower in iVSD hearts compared to control hearts. GAPDH served as a control. (A) *CTGF* and *AXL* relative mRNA levels were significantly lower in iVSD compared to control subjects. *GAPDH* served as a control. Data presented as means ± SD. The Student’s *t*-test was performed to evaluate statistical significance, iVSD group: n = 20, control group: n = 6, **p<0.01.

Since *YAP1* was significantly reduced in iVSD hearts, we analyzed the expression of its downstream targets. Here we measured gene expression levels of two frequently used markers of YAP1 activity: *CTGF* and *AXL* [10–12]. As expected, the expression of both CTGF and AXL were downregulated (Fig 4). These results showed that both *YAP1* and its downstream targets were reduced in iVSD hearts compared to normal hearts.

### Proliferation activity of iVSD cardiomyocytes and non-cardiomyocytes by flow cytometry analysis

**Fig 5:**
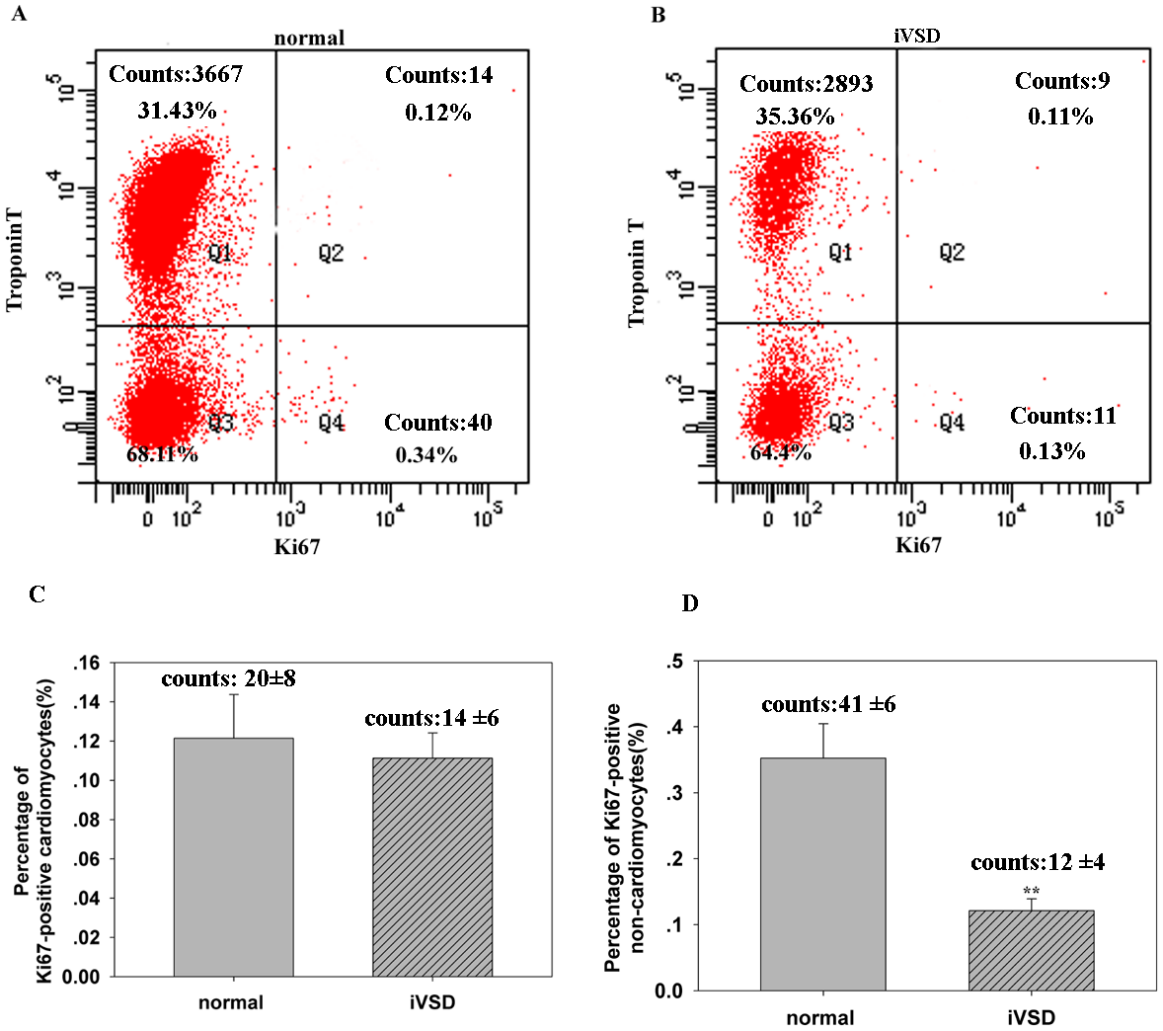
Proliferation rate of cardiomyocytes in iVSD hearts is comparable to control hearts, while proliferating non-cardiomyocytes are significantly reduced in iVSD hearts. Flow cytometry analysis showed that the percentage of Ki67-positive cardiomyocytes was similar in hearts of iVSD patients compared to control hearts and that Ki67-positive non-cardiomyocytes were significantly reduced in iVSD patients compared to control hearts. (A) Representative Ki67-positive cells in control hearts. (B) Representative Ki67-positive cells in iVSD hearts. (C) Quantification of Ki67-positive cardiomyocytes (D) Quantification of Ki67-positive non-cardiomyocytes (iVSD group: *n* = 20; control group: n = 6). Data presented as means ± SD; **p<0.01.

Because YAP1 has been shown to affect cardiac cell proliferation and contributes to ventricular septal closure in mice, we compared cardiac cell proliferation in iVSD hearts to control hearts to determine whether YAP1 was still functional in the postnatal period. We chose to analyze Ki67 expression because Ki67 is present during all active phases of the cell cycle (G1, S, G2 and mitosis), which makes it an excellent marker of proliferation. Using flow cytometry, the number of proliferating cardiomyocytes was assessed in both groups. Based on the combination of Ki67- and troponin-positive staining, the number of proliferating cardiomyocytes (Fig 5) were similar in the iVSD group compared to the control group (14 ±6 in iVSD vs. 20±8 in control group; *p = ns*). A total of 10,124 ±2452 cells were counted from the iVSD group (n = 20), and 12,344 ±3463 cells were counted from the normal group (n = 6) (The percentage of Ki67-positive cardiomyocytes in iVSD group and control group were 0.11± 0.02 and 0.12 ± 0.02, respectively; *p* = ns). For Ki67-positive non- cardiomyocytes, in the iVSD group 12 ±4 and normal control 41 ±6 cells were counted (*p*<0.01) (The percentage of Ki67-positive non-cardiomyocytes in iVSD group and control group were 0.13± 0.02 and 0.38 ± 0.06, respectively; *p*<0.01). The results indicate that YAP1 expression primarily affected the non- cardiomyocyte cell proliferation activity in the postnatal iVSD heart.

### Proliferation activity of iVSD cardiomyocytes and non-cardiomyocytes by confocal microscopy

**Fig 6:**
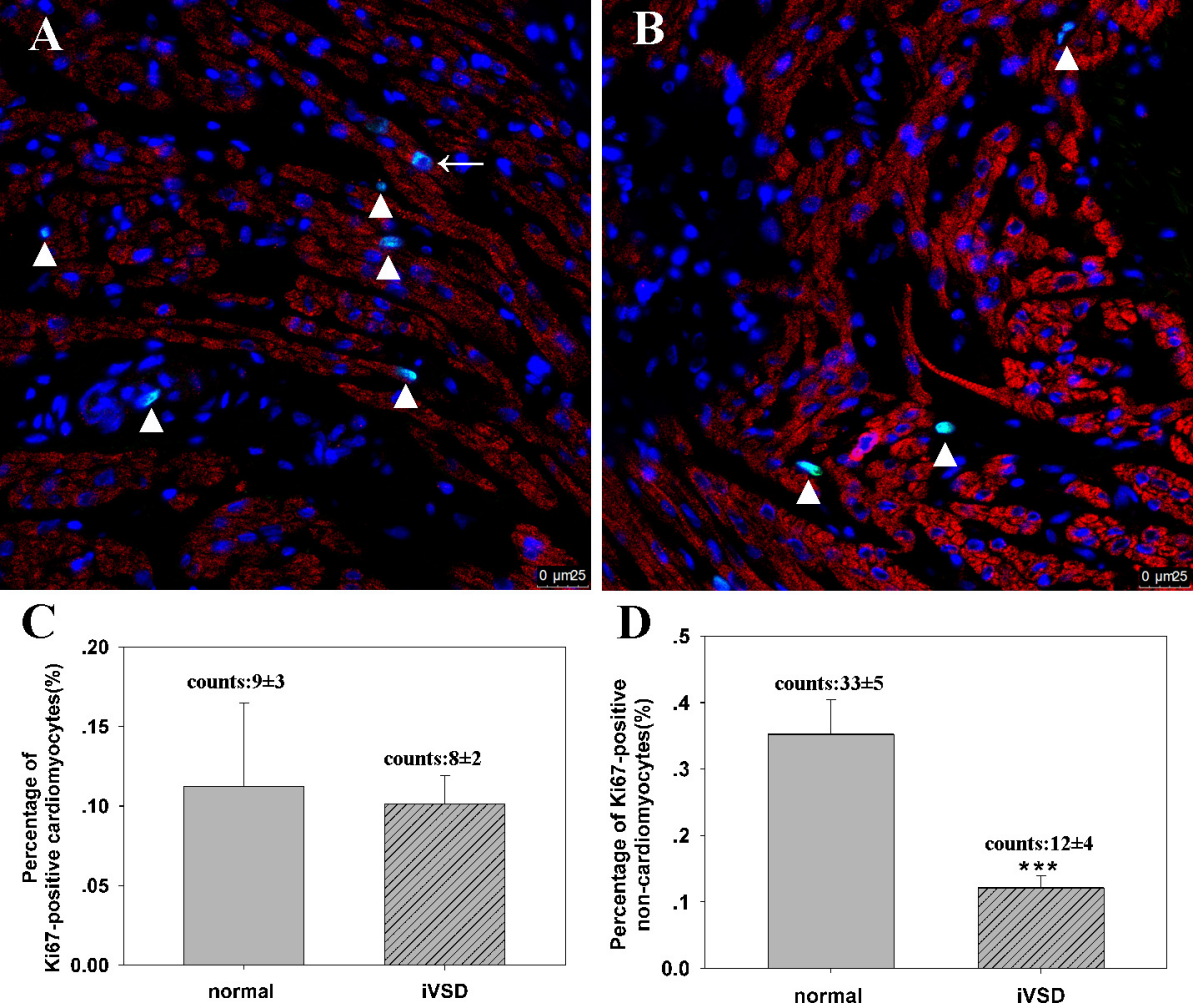
Proliferation of iVSD cardiomyocytes analyzed *via* confocal microscopy. Confocal microscopy of tissue sections using Ki67 and troponin indicate that non-cardiomyocyte cell proliferation is decreased in iVSD tissues compared to controls. (A) Representative Ki67-positive cells in normal hearts. (B) Representative Ki67-positive cells in iVSD hearts. (C) Quantification of Ki67-positive cardiomyocytes. (D) Quantification of Ki67-positive non-cardiomyocytes (iVSD group: n = 20; control group: n = 6. Data presented as means ± SD; ***p<0.001. Cardiac troponin T (red), Ki67 (green), and DAPI (blue) staining are shown. Arrow indicates proliferating cardiomyocytes and the triangle indicates non-cardiomyocytes.

To confirm the flow cytometry data, we quantified proliferating cardiomyocytes in tissue sections using confocal microscopy. Data revealed that the percentage of Ki67-positive cardiomyocytes in normal and iVSD hearts were 0.11 ± 0.05 (number of Ki67- positive cardiomyocytes: 9±3, number of total counted cells: 9782±1873, n = 20) and 0.10 ± 0.02 (number of Ki67- positive cardiomyocytes: 8±2, number of total counted cells: 9886±1451, n = 6), respectively (*p* = ns), while the percentage of Ki67-positive non-cardiomyocytes in both groups were 0.35 ± 0.05 (number of Ki67- positive non-cardiomyocytes: 33±5) and 0.12 ± 0.02 (number of Ki67- positive non-cardiomyocytes: 12±4), respectively (*p* < 0.01; Fig 6). Proliferating cells measured with staining correlated well with flow cytometry data from isolated cardiac cells, confirming that non-cardiomyocyte proliferation activity was reduced significantly in iVSD patients.

## Discussion

Isolated VSD is the most commonly occurring CHD, affecting 2 of every 1,000 live births, and accounting for approximately 20% of all forms of CHD. VSD and iVSDs associated with other major defects account for over half of the patients with congenitally malformed hearts [13]. Therefore understanding iVSD is essential for uncovering the etiology of CHDs. Unfortunately because no iVSD animal model exists and decedent fetal tissue of human iVSD is unavailable, obtaining data from human postnatal iVSD tissue may help uncover the underlying mechanisms. The significant findings of this study were that 1) YAP1 and its downstream targets were significantly decreased and 2) non-cardiomyocyte cell proliferation was significantly decreased in iVSD human samples compared to control subjects.

Closing an open anatomical structure is a recurrent theme in mammalian embryology, and this process plays an integral role not only in the development of ventricular septum, but also in the palate, neural tube, urethra, diaphragm and eye [14]. Studies of metazoan developmental genetics suggest that evolution employs a relatively small number of signaling pathways to control a wide range of embryological processes [4]. Thus understanding how iVSD occurs could extend research into understanding cleft palate, neural tube malformation as well as other anatomical structures that require closure. This closure process includes three events: PCP, EMT, and cell proliferation, of which the latter two are directly regulated by YAP1 [5,6]. PCP may be indirectly regulated by YAP1 coordinating with Wnt signaling [14]. Thus, investigating the expression of YAP1 in iVSD human samples is essential.

YAP1 expression is significantly altered in iVSD hearts. Both *YAP1* mRNA and protein in iVSD hearts is only one-third that of control hearts. The Hippo signaling pathway is an evolutionarily conserved mechanism that controls cell proliferation and organ size by phosphorylating its key effector, the transcriptional coactivator YAP. This phosphorylation prevents YAP from entering nucleus where it associates with DNA-binding transcription factors to drive cell growth-related gene expression [15, 16]. Immunofluorescence analysis revealed YAP1 was chiefly expressed in the nuclear, indicating potential transcriptional activity. Because live cells are required for testing PCP and EMT, normal hearts specimens stored in destructive liquid nitrogen could not be used to examine PCP and EMT events. Therefore, to verify the functionality of YAP1, we measured cell proliferation.

Historically, the human heart has been considered a post-mitotic organ. However recent research suggests that human cardiomyocyte proliferation after birth contributes to the postnatal heart growth [17–19], making the study of myocyte proliferation possible. We found that the differences in proliferation of cardiomyocytes between iVSD and control hearts were subtle but that proliferative non-cardiomyocytes were significantly reduced in iVSD. This may be explained by the rigid structure of cardiomyocytes [20] that lose the ability to divide gradually with age [19]. Nevertheless data from non-cardiomyocytes suggested YAP1 was still functional in postnatal closure of iVSD. In clinical observations, spontaneous closure of VSD in postnatal period is possible if the diameter of defect is less than 0.5cm, the mechanisms of which include marginal fibrosis induced by hemodynamic changes; adherence of the septal leaflet of the tricuspid valve to the defect, often forming a tricuspid tissue pouch; and muscular hypertrophy [21]. However larger VSDs (diameter>0.5cm) cannot be closed spontaneously, and current studies suggest that YAP1 may be a target that could be manipulated to allow closure of larger VSDs.

We report that cell proliferation and YAP1 expression in hearts of iVSD and normal patients, indicating that YAP1 might contribute to the embryonic formation of human iVSD and retain its functionality during the postnatal period. In recent years, several studies have demonstrated that YAP1 is modulated by not only the Hippo pathway, but also by other several signal transduction pathways, therefore YAP1 interacts with many downstream and upstream signaling proteins [22–24]. As such, YAP1 is involved in a complex signal transduction network; therefore, a systemic analysis of this network will further define the role of YAP1 during ventricular septum development.

## Supporting Information

S1 Table Clinical Features of iVSD. (XLS)
